# Advances in Agricultural Applications of Lignans

**DOI:** 10.1002/cbdv.71862

**Published:** 2026-10-21

**Authors:** Kaida Li, Huawen Wang, Xiangjie Luo, Feng Hao, Kun Wei, Le Cai

**Affiliations:** ^1^ Yunnan University Kunming People's Republic of China; ^2^ School of Chemical Science and Technology Yunnan University Kunming People's Republic of China; ^3^ Key Laboratory of Medicinal Chemistry for Natural Resource Yunnan University Kunming People's Republic of China

**Keywords:** agricultural bioactivity, botanical pesticide, feed additive, lignans

## Abstract

Despite well‐documented pharmacological profiles of lignans in human therapeutics, their agricultural and veterinary potential remains fragmented without unified collation. This systematic review synthesizes literature published from 2001 to 2026, categorizing lignan compounds by carbon‐skeleton classification frameworks. Representative classic lignans, neolignans, oligolignans, and complex lignans exhibit diverse bioactivities, including antifungal, antibacterial, plant‐growth‐regulating, herbicidal, insecticidal, and acaricidal properties. We compiled quantitative bioassay metrics including half‐maximal effective concentration (EC_50_), median lethal concentration (LC_50_), and minimum inhibitory concentration (MIC), alongside livestock production indices to compare agricultural and veterinary performance. Structure‐activity relationships and molecular mechanisms of key lignan scaffolds are analyzed, and research bottlenecks for each subtype are highlighted. Prospects are outlined for developing lignan‐derived green botanical pesticides and natural antibiotic‐replacing feed additives. This review delivers systematic data and theoretical reference for further exploitation of lignan‐based agrochemical leads and livestock antibiotic alternatives.

## Graphical abstract

This graphical abstract presents lignans as green alternatives to chemical pesticides and antibiotics for agriculture and veterinary use. It illustrates their applications in plant protection (antifungal, insecticidal, herbicidal, fruit preservation) and aquaculture, classifies lignan subtypes from classic to complex forms, highlights limitations like poor solubility and unclear targets, and proposes rational modification and microcapsule formulations as future directions.

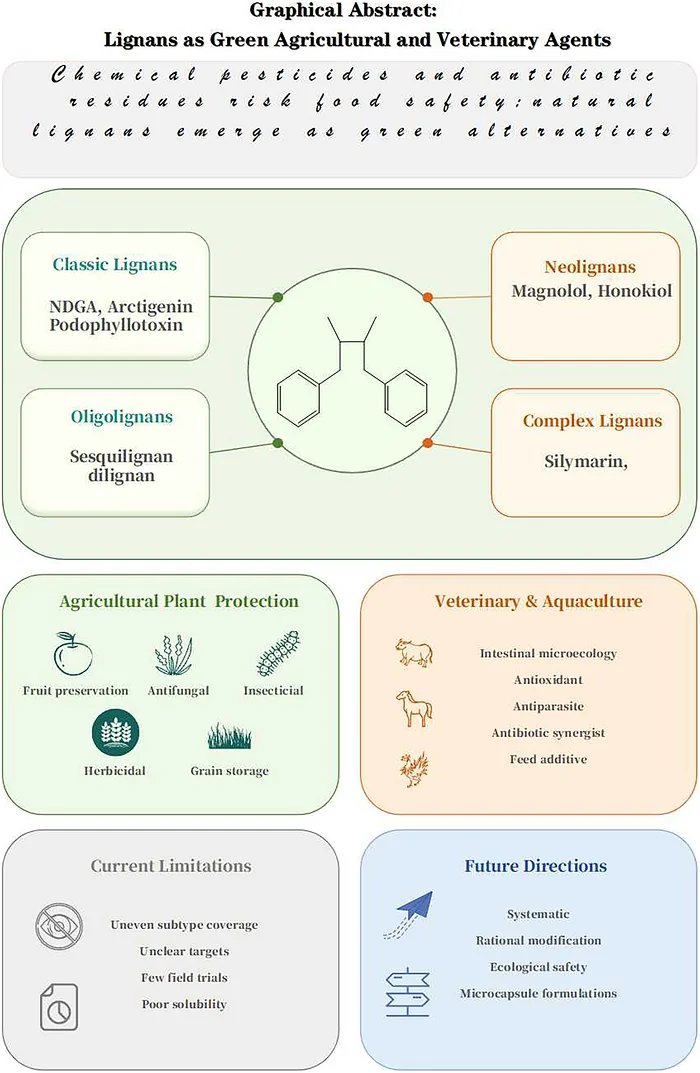

## 1 Introduction

Lignans are formed by oxidative coupling of two C6‐C3 phenylpropane units, and their classification is determined by the coupling position of carbon skeletons [1]. According to the classical phytochemical classification criteria [2], lignans are divided into four major types: (1) Classic lignans connected by β─β′ bonds (including dibenzylbutane, dibenzylbutyrolactone, arylnaphthalene lignan and other seven subclasses); (2) neolignans connected by non‐β─β′ bonds (benzofuran, benzodioxane, biphenyl three subtypes) [3]; (3) oligolignans polymerized by three or more phenylpropane monomers [4]; (4) complex lignans hybridized with flavonoids, coumarins and other secondary metabolites [5]. Lignans are widely distributed in Magnoliaceae, Cupressaceae, Linaceae, Podophyllaceae, Lauraceae, and other plants [6], and most of them have multiple phenolic hydroxyl groups, endowing them with excellent antioxidant [7], antibacterial, and anti‐inflammatory properties [8].

At present, most research on lignans focuses on human medical fields such as anti‐tumor [9], liver protection [10], and cardiovascular protection [11], while the research on agricultural plant protection and veterinary breeding is scattered and lacks systematic classification combing. In agricultural production, long‐term single use of chemical pesticides leads to widespread pest and pathogen resistance [12], and chemical residues threaten food safety and the ecological environment [13]. Environment‐friendly microbial and botanical biopesticides provide feasible mitigation pathways for such risks, as illustrated by screening of local high‐virulence *Bacillus thuringiensis* isolates for lepidopteran pest control [14]. In parallel, a new wave of structurally diverse green agrochemical candidates has recently been developed through natural‐product‐based and scaffold‐hopping strategies, including natural pyrethroid derivatives from pyrethrum residues with reduced toxicity to aquatic organisms [15], pentafluorobenzene‐type insecticides targeting insect cuticular proteins [16], sulfone‐type [17], thioether‐type [18], acylhydrazine‐type [19], and 1,3,4‐oxadiazole‐type derivatives [20], collectively illustrating the current trend toward low‐toxicity, low‐residue pesticide innovation. In the breeding industry, the global restriction and prohibition of antibiotic additives makes the development of natural green substitutes an urgent demand [21]. Lignans, as plant‐derived active substances with low toxicity and easy biodegradation, have become important lead compounds for new green pesticides and functional feed additives [22].

Based on the existing domestic and foreign literatures from 2001 to 2026, this review takes the chemical skeleton classification as the main line, systematically summarizes the agricultural and veterinary bioactivities of each subtype of lignans, sorts out the test data, action mechanisms, and structure–activity rules of representative compounds, analyzes the existing bottlenecks in current research, and puts forward feasible research directions, so as to provide a systematic reference for the subsequent excavation and structural optimization of lignan agricultural and veterinary lead compounds.

The classification framework adopted in this review is summarized in Figure 1.

**FIGURE 1:**
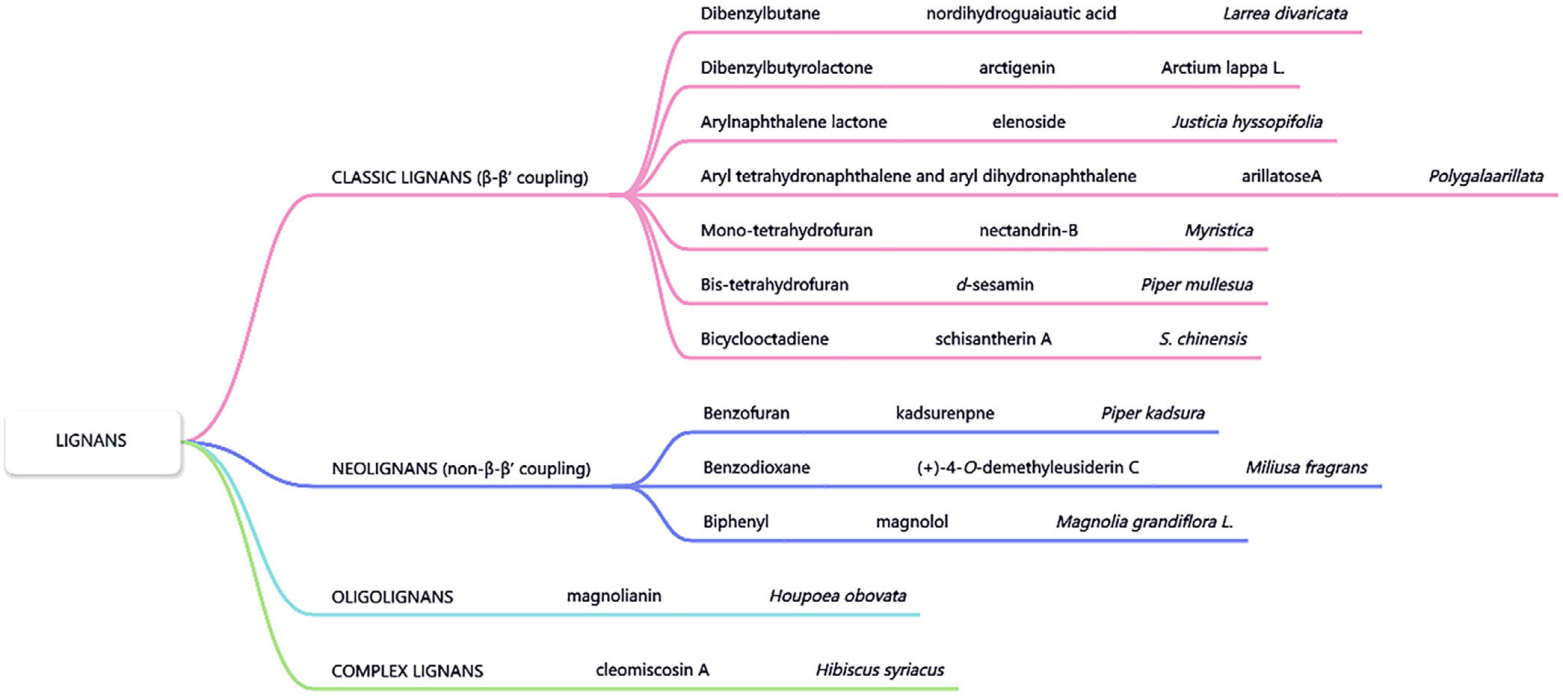
Classification of lignans by carbon‐skeleton types with representative active compounds and their botanical sources.

## 2 Methodology

This work is a systematic narrative review focusing on agricultural and veterinary applications of lignan natural products. Literature retrieval was performed across Web of Science, Scopus, Google Scholar, and CNKI databases, covering publication years from 2001 to 2026. Search terms included combinations: “lignan”, “neolignan”, “botanical pesticide”, “antifungal”, “insecticidal”, “herbicidal”, “feed additive”, “veterinary”, and “structure–activity relationship”. Inclusion criteria: peer‐reviewed original‐research articles and comprehensive reviews reporting lignan/neolignan bio‐assays targeting plant‐protection or livestock‐breeding scenarios.

Exclusion criteria: papers only focusing on human‐medical pharmacology without agro‐veterinary phenotypic data, conference abstracts without full dataset. Extracted information covers lignan carbon‐skeleton classification, source organism, quantitative bioassay values (EC_50_, LC_50_, MIC), in vivo pot / field trial outcomes, animal‐production‐performance indices, structure‐activity observations and reported molecular targets. Literature were grouped and summarized by lignan structural sub‐classes; research gaps and bottlenecks for each subtype were documented.

Compared with previously published lignan‐related reviews which predominantly focused on human pharmacological properties (anti‐cancer, hepatoprotective effects), this review possesses distinct incremental contributions. Existing reviews only provide fragmentary descriptions of partial agricultural or veterinary findings, without systematic sorting according to lignan carbon skeleton. In contrast, this work unifies plant‐protection and livestock‐veterinary perspectives; quantitatively compiles bioassay datasets; highlights research imbalance across structural subclasses; strictly differentiates pure compounds versus crude extracts; and comprehensively analyzes bottlenecks including molecular‐mechanism ambiguity, safety risks, formulation challenges, and translational limitations for lignan‐derived agro‐veterinary leads. This fills the gap for a dedicated comprehensive review covering both agricultural and veterinary utilization of lignans.

We classified the reported bioactivities into four evidence levels: (1) In vitro level—isolated pathogen/insect/cell assays; (2) in vivo‐laboratory level—whole‐organism tests under controlled lab conditions (e.g., potted seedlings, caged insects); (3) animal feeding level—livestock or aquaculture feeding trials; and (4) field level—open‐field or commercial‐scale validation. Throughout the manuscript, we have strictly used these terms to distinguish preliminary observations from confirmed agricultural applications.

## 3 Classic Lignans

Classic lignans are dimeric phenylpropane metabolites linked by β─β′ carbon‐carbon bonds, which are further divided into seven subclasses according to ring formation modes and substituent structures. A large number of studies have confirmed that this type of lignans have diverse agricultural and veterinary potentials such as fruit ripening regulation, bacteriostasis, insect killing, antiparasitic, and feed antioxidation.

### 3.1 Dibenzylbutane Lignans

Dibenzylbutane lignans represented by nordihydroguaiaretic acid (NDGA) **(1)** and secoisolariciresinol diglucoside (SDG) **(2)** are the most thoroughly studied subtype in this class, covering multiple agricultural and veterinary application scenarios (Figure 2).

**FIGURE 2:**
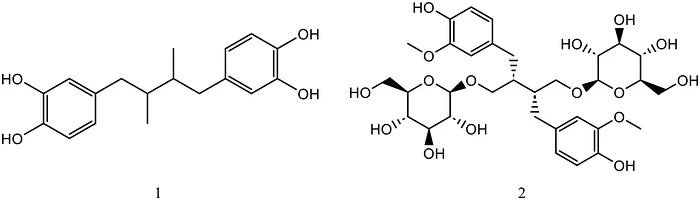
The structures of **1** and **2**.

In agricultural field, NDGA is a specific inhibitor of ABA synthetase NCED, which can precisely regulate the ripening process of various fruits. In 2026, Dai et al. used [23] vacuum infiltration to treat multiple climacteric fruits with NDGA. The results showed that NDGA significantly down‐regulated the expression of ethylene synthesis genes *SlACO1* and *SlACS2*, inhibited endogenous ABA accumulation, delayed fruit color change and softening, and had broad‐spectrum fresh‐keeping effects on tomato, apple, banana, mango and apricot. In 2024, Azuara et al. [24] carried out field experiments on Verdejo grapes. Spraying 100 µM NDGA before veraison delayed grape harvest by 4 days, balanced fruit sugar‐acid ratio, increased total polyphenol index by 19%, and improved wine flavor quality without affecting tree vigor and yield. In 2023, Wu et al. [25] adopted TMT quantitative proteomics to reveal that NDGA regulated multiple pathways such as cell wall degradation and antioxidant metabolism in cherry tomatoes, and delayed ethylene peak occurrence by 2 days. Related studies on melon seedlings, strawberries, sweet cherries, kiwifruit, figs, and citrus also confirmed that NDGA can inhibit NCED to block ABA signal, and has application value in pre‐harvest quality regulation and post‐harvest fresh‐keeping of fruits [26, 27, 28, 29, 30, 31, 32]. In addition, NDGA showed potent lipoxygenase (LOX) inhibitory activity in vitro (92.8% inhibition at 100 µM) [33], suggesting a potential application in delaying lipid peroxidation during grain and oil crop storage, pending formulation and post‐harvest validation. Moreover, a recent comprehensive review systematically collated 193 natural, semisynthetic and synthetic molecules with plant growth‐promoting activities reported between 2005 and 2026, covering fruit ripening, seed germination and plant resistance enhancement, thereby providing a systematic molecular template for plant‐growth‐regulator discovery in which lignan‐type regulators such as NDGA occupy a defined niche [34]. Separately, a field‐scale trial on Verdejo grapes demonstrated that pre‐veraison spraying with 100 µM NDGA delayed harvest by 4 days under open‐field conditions. For other fruit species, only laboratory‐ or controlled‐environment data are currently available.

In terms of antibacterial and herbicidal agricultural activity, meso‐dihydroguaiaretic acid (meso‐DGA) **(3)** has broad‐spectrum activity. At 1 mM, the root growth inhibition rate of Italian ryegrass reaches 69%, with an LC_50_ of 5.12 × 10^–^^5^ M against Culex pipiens larvae; at 0.25 mM, it has inhibition rates of 32.0%–53.7% against *Alternaria alternata*, *Fusarium solani*, and other plant pathogenic fungi. Its antibacterial spectrum is specific to Gram‐positive bacteria such as *Staphylococcus aureus* and *Bacillus subtilis*, with MIC as low as 1.56 Mm [35]. QSAR analysis clearly indicates that introducing electron‐withdrawing groups (e.g., F, Cl) at the 7‐phenyl ring of DGA significantly enhances antifungal activity against *A. alternata* Japanese pear pathotype, with representative derivatives **4a** (3‐F, EC_50_ = 20.4 µM), **4b** (3‐Cl, 24.6 µM), and **4c** (3,5‐difluoro, 15.5 µM) showing high potency, whereas electron‐donating groups (e.g., ─CH_3_, ─OCH_3_) drastically reduce activity, as exemplified by the 3,4‐dimethyl derivative **4d** (EC_50_ = 235 µM), the least active among the series. Positional effects demonstrate that meta‐substitution (**4a**, **4b**) is most favorable, followed by ortho (**4e**, **4f**) and para (**4g**, **4h**). Steric parameters further reveal that smaller substituents are beneficial, as fluorine (**4a**) outperforms the bulkier chlorine (**4b**). Notably, the unsubstituted phenyl derivative **4i** (EC_50_ = 33.6 µM) already exhibited about 2‐fold higher activity than natural (+)‐DGA (**3**, EC_50_ = 71.8 µM), confirming the importance of the phenyl ring itself. Guided by these structure–activity relationships, the 3,5‐difluorophenyl derivative (**4c**) exhibits the highest activity, with an EC_50_ value of 15.5 µM—an approximately 4.6‐fold improvement over compound **3**—making it a promising lead compound for agricultural antifungal development. In addition, for the apple pathotype of *A. alternata*, compound **4j** (3‐chloro‐4‐hydroxyphenyl) shows specific potency (EC_50_ = 36.9 µM); meanwhile, the 3‐hydroxy‐4‐alkoxy derivatives (**4k‐4m**), though less antifungal, unexpectedly display antimelanogenic (whitening) activity, thereby broadening the potential applications of this lignan scaffold [36]. Extracts containing NDGA from *Chromolaena hookeriana* have strong DPPH free radical scavenging ability (>90%) and antibacterial activity against *S. aureus*, with low toxicity to Artemia, which can be developed as agricultural bacteriostatic agents [37] (Figure 3).

**FIGURE 3:**
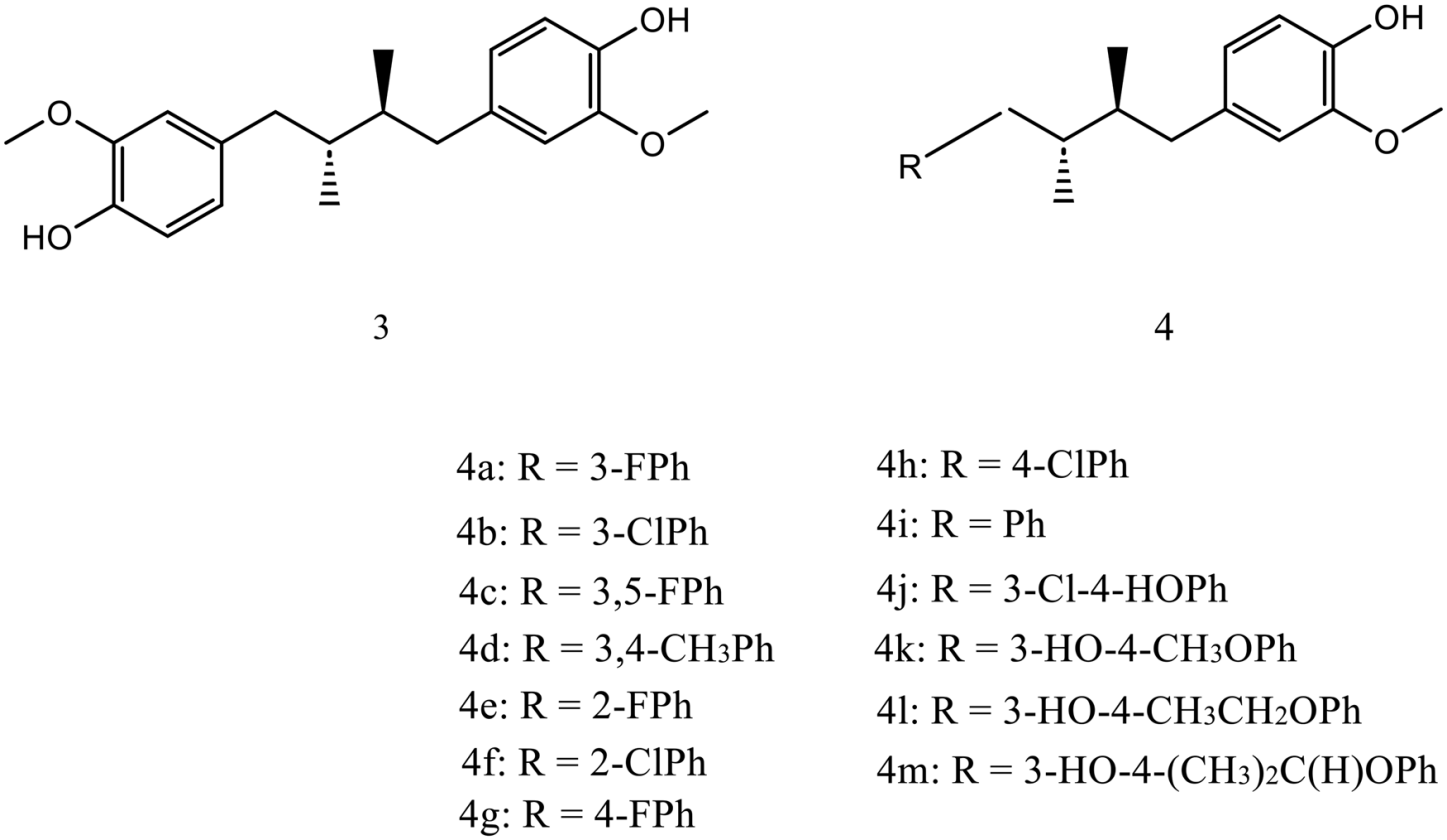
The structures of **3** and **4**.

In veterinary applications, SDG rich in flaxseed has multiple functions such as intestinal regulation, antioxidation and bone health promotion. In 2024, Jewell et al. [38] carried out 28‐day feeding experiments on dogs. Adding fiber raw materials rich in SDG significantly increased butyrate content in feces, regulated intestinal microbial metabolic mode, and its metabolites enterodiol and enterolactone had anti‐inflammatory and antioxidant effects. In 2024, Iqlima et al. carried out 30‐day fermentation experiments on oil palm empty fruit bunches. Inoculating with the isolated lignocellulolytic fungi *Lasiodiplodia theobromae* and *Acremonium* sp. significantly reduced the Neutral Detergent Fiber (NDF) and lignan levels of empty fruit bunches, enhanced their in vitro true digestibility (IVTD) and digestible NDF (dNDF), and the results suggested that these fungi could degrade the lignocellulose complex and effectively increase the digestibility of oil palm empty fruit bunches as ruminant feedstuff [39]. In 2017, Figueiredo et al. [40] supplemented SDG to lactating rats, and the bone mineral density of offspring increased significantly in adulthood through estrogen receptor regulation. Feeding experiments on laying hens showed that SDG in flaxseed can be metabolized and enriched in egg yolk, change n‐6/n‐3 fatty acid ratio of egg yolk, and regulate animal estrogen level [41]. Systematic review by Imran et al. [42] confirmed that SDG has veterinary potentials including liver protection, kidney injury intervention, reproductive regulation and lipid metabolism improvement. In rumen perfusion experiments with Huainan goats, SDG was converted into enterodiol and enterolactone by rumen microorganisms, and was associated with improved protein synthesis efficiency and volatile fatty acid yield under the experimental conditions tested [43]. These findings suggest its potential as a ruminant feed additive, although multi‐breed and long‐term feeding validation is still required [43]. In addition, SDG and its aglycone secoisolariciresinol have equivalent antioxidant capacity to BHT in rapeseed oil storage test, and can replace synthetic antioxidants in livestock and aquatic feed to prevent feed rancidity [44]. This potential of plant‐derived bioactive compounds extends beyond antioxidative preservation. In ruminant feeding, tannin‐rich tropical legumes such as *Indigofera zollingeriana* and *Calliandra calothyrsus* have been combined with sorghum as locally available feed alternatives; an in vitro rumen fermentation study by Dahlya et al. [45] demonstrated that the 60% sorghum–40% *I. zollingeriana* combination improved dry matter, organic matter and fiber‐fraction digestibility as well as VFA and NH_3_ production, while the 60% sorghum–40% *C. calothyrsus* combination achieved the highest microbial protein synthesis (26.93 mg/mL) and tended to reduce methane and total gas production, without significantly increasing rumen gas emissions across treatments. These findings indicate that legume‐derived bioactive constituents, mainly tannins, can modulate rumen microbial ecology, thereby supporting economical and sustainable ruminant production and complementing synthetic antioxidant replacement in feed.

### 3.2 Dibenzylbutyrolactone Lignans

Arctigenin (**5**) is the representative compound of this subtype, with reported insect‐cell cytotoxicity, aquatic antiparasitic and antiviral activities in laboratory settings (detailed below).

In agricultural insecticidal field, (8R,8′*R*)‐*trans*‐arctigenin (**5**) has stereospecific selective cytotoxicity to insect cells. Its IC_50_ against *Spodoptera frugiperda* Sf9 cells and *Aedes albopictus NIAS‐AeAl‐2* cells are 6.7 and 0.73 µM, respectively, far superior to other stereoisomers (**6**, **7**, **8**). Structural modification on the aromatic ring of the (8R,8′*R*)‐*trans* scaffold found that 3‐iodo (**9b**), 4‐iodo (**9c**) and 3,4‐methylenedioxy (**9d**) derivatives maintain equivalent activity against *Sf9* cells, and the 4‐hydroxy derivative (**9a**) shows comparable toxicity to mosquito *NIAS‐AeAl‐2* cells. Thiono derivatives including 4‐iodo thiono (**10a**) and 3,4‐methylenedioxy thiono (**10b**) analogs also retain potent insect cell cytotoxicity, while all enantiomers of these active derivatives (**11a**, **11b**, **11c**) show decreased insecticidal activity. Its toxic action against insect cells is related to the regulation of ribosomal RNA metabolism via upregulating *28S rRNA* expression, without inducing DNA degradation [46]. In 2023, Xu et al. isolated a dibenzylbutyrolactone lignan (**12**) from Cinnamomum camphora, with LC_50_ of 0.106 µg/mL against *Culex pipiens quinquefasciatus* fourth‐instar larvae, showing mosquito larvicidal activity [47]. (−)‐Kusunokinin (**12**) extracted from Aristolochia malmeana showed dose‐dependent toxicity to *Anticarsia gemmatalis* fourth‐instar larvae in laboratory bioassays, and hinokinin derived from the same skeleton exhibited synergistic effects with pyrethroids under the same experimental conditions [48]. In laboratory feeding assays, yatein (**13**) and hibalactone from *Juniperus sabina* exhibited antifeedant and stomach toxicity against *M. separata* and *P. xylostella*, and were also associated with inhibited growth and reproduction under the experimental conditions reported [49] (Figure 4).

**FIGURE 4:**
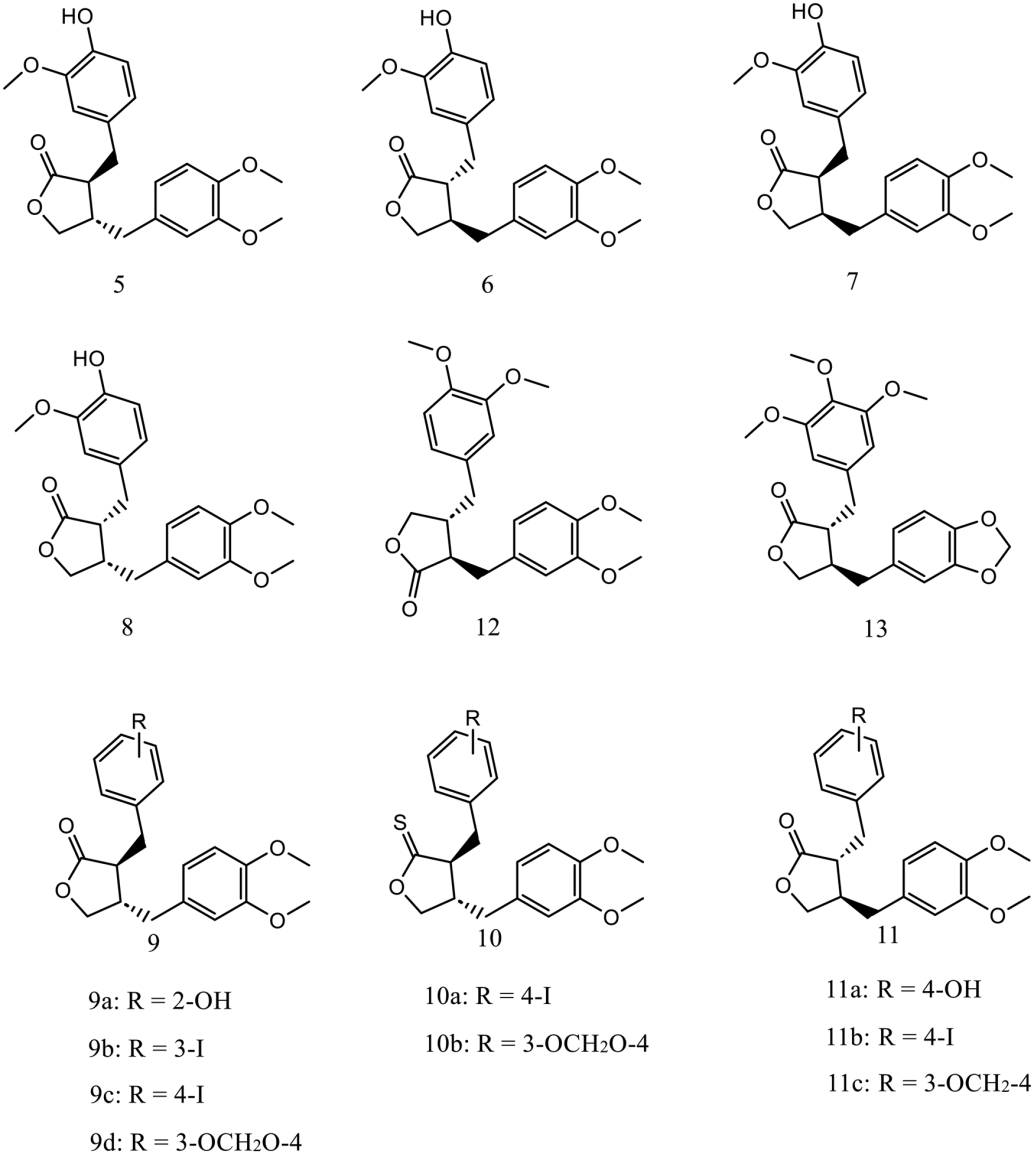
The structures of **5–13**.

In the veterinary aquaculture field, arctigenin (**14**) and its derivatives have excellent anti‐monogenean and anti‐SVCV activity. A series of arctigenin ether and heterozygous imidazole derivatives designed by Hu et al. have EC_50_ as low as 1.52 mg/L against Dactylogyrus intermedius, with therapeutic index up to 19.36, far safer than praziquantel [50]. In 2024, Tan et al. synthesized arctigenin ester derivatives targeting myosin of Gyrodactylus kobayashii, among which compound **15** had an EC_50_ of 0.4 mg/L and inhibited parasite energy metabolism by destroying body wall structure [51]. In terms of aquatic antiviral activity, arctigenin imidazole octyloxy derivative **16** has an IC_50_ of 0.095 µg/mL against SVCV, which can inhibit ROS burst and cell apoptosis of carp epithelial cells, and has application prospect in the prevention and control of spring viremia of carp [52] (Figure 5).

**FIGURE 5:**
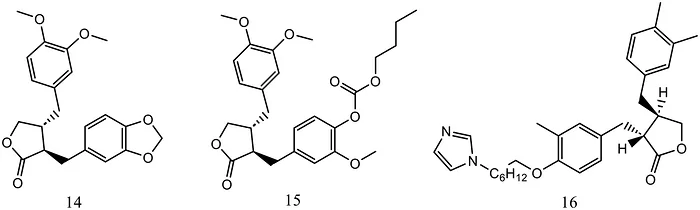
The structures of **14–16**.

The insecticidal activities of dibenzylbutyrolactone lignans are primarily derived from in vitro cell‐based or laboratory larval bioassays; no field trial data have been reported for this subtype to date.

### 3.3 Arylnaphthalene Lactone Lignans

Podophyllotoxin (PPT **17**) and its derivatives, helioxanthin (**18**) and taiwanin E (**19**), are typical compounds of this subtype, which are the most widely studied lignan lead compounds for agricultural insecticidal and antifungal development (Figure 6).

**FIGURE 6:**
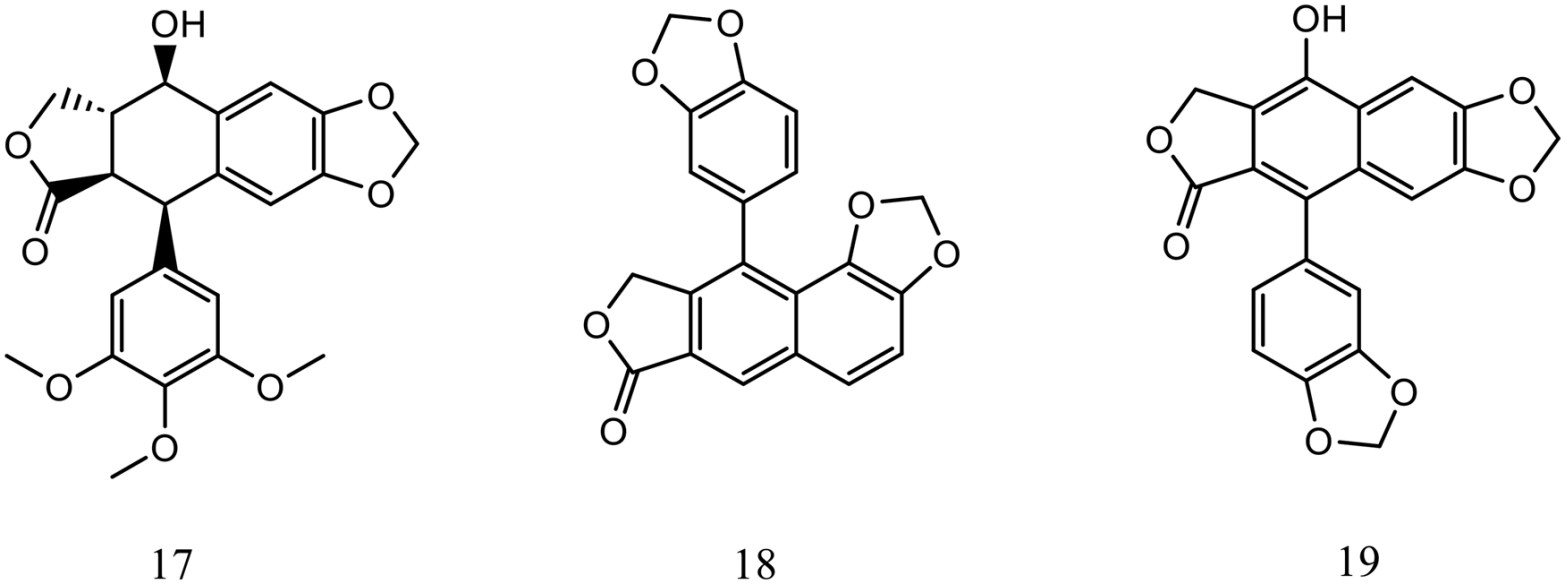
The structures of **17–19**.

In agricultural insecticidal and acaricidal fields, a large number of structural modification studies have proved that PPT derivatives have significant stomach toxicity, antifeedant and growth regulation activities against lepidopteran pests such as *M. separata* and *Pieris rapae*. A variety of halogenated, isoxazoline fused, oxime sulfonate, hydrazone and D‐ring open‐loop PPT derivatives synthesized from 2008 to 2026 have corrected mortality of 50%–72.4% against *M. separata* at 1 mg/mL, which is better than positive control toosendanin (48.3% or 43.3%) in the same bioassay. Notable examples include oxime sulfonate derivatives **20, 21, and 22** (corrected mortality 62.1%, 65.5%, and 62.1%, respectively), hydrazone derivatives **23** and **24** (63.3% each), and D‐ring opened derivative **25** (65.5%). These compounds consistently outperformed toosendanin (48.3% or 43.3%) in the same bioassay [22, 53, 54, 55, 56, 57, 58, 59, 60, 61, 62, 63, 64]. Structure–activity analysis indicated that E‐ring halogenation, C‐4 esterification, and heterocyclic fusion could improve activity in laboratory acaricidal bioassays; several derivatives showed EC_50_ values as low as 1/13 of that of the parent PPT against *T. cinnabarinus* under these experimental conditions [65]. Helioxanthin (**27**) separated from *Taiwania flousiana* showed larvicidal activity in laboratory bioassays with an LC_50_ of 0.60 mg/L against *Aedes aegypti* second‐instar larvae, and mitochondrial VDAC protein was identified as a putative target. Its sulfonylated derivative **28** exhibited improved solubility and lower toxicity to bees and non‐target plants in laboratory assessments [34, 66]. Multi‐omics research in 2026 further clarified that **27** inhibits ferrochelatase to block heme synthesis, induce mitochondrial collapse and kill mosquito larvae, providing a new target for pesticide development [67] (Figure 7).

**FIGURE 7:**
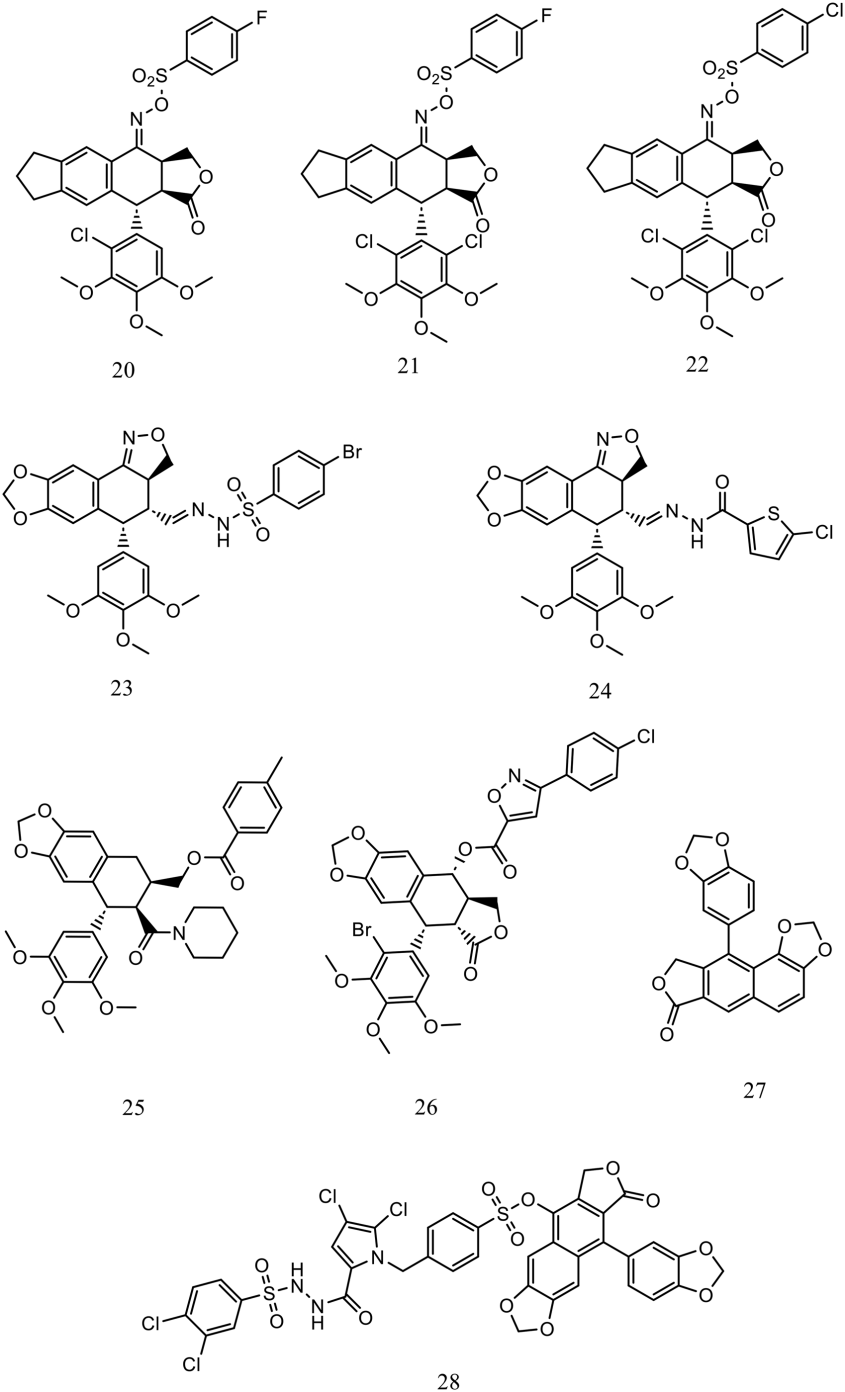
The structures of **20–28**.

In a field trial against rice blast, a podophyllotoxin‐rich (**17**) extract from *P. hexandrum* showed control efficacy equivalent to tricyclazole and reduced rice yield loss by 47.6% compared to the blank control [68]. Molecular docking suggested that podophyllotoxin may interact with the ToxB effector protein, but this binding mode requires experimental validation. In addition, the lignans of this subtype have multitarget binding affinity against *Spodoptera litura*, such as amentoflavone, and have the potential of multisite insecticidal activity [65, 69].

In line with these arylnaphthalene leads, chemical modification of the arylnaphthalene lactone lignan taiwanin E (19) from the bark of *T. flousiana* improved its solubility and insecticidal activity, and the optimized derivative E13 displayed potent larvicidal activity against *Armigeres subalbatus* larvae with confirmed environmental safety in bee‐toxicity tests, as validated by enzyme assays, molecular docking and DFT calculations [34]. In parallel, essential oils from the heartwood of *T. flousiana* showed broad‐spectrum insecticidal activity against multiple pest species with low toxicity to non‐target organisms (*Nicotiana tabacum*, *Danio rerio*, and *Apis mellifera*), indicating that this endangered conifer represents a valuable source of both lignan and non‐lignan insecticidal principles [70].

Except for the single field trial of podophyllotoxin against rice blast, all insecticidal/acaricidal data for arylnaphthalene lignans are derived from laboratory bioassays. The promising in vitro potency of helioxanthin awaits further greenhouse and field evaluation.

### 3.4 Aryl Tetrahydronaphthalene and Aryl Dihydronaphthalene Lignans

Aryl‐tetrahydronaphthalene‐type lignans are the most prevalent among naturally occurring lignans, as exemplified by the representative compound 1‐isolariciresinol (**29**), as well as by arillatose A (**30**)—a glycolipid derived from the roots of *Polygala arillata*, a traditional Chinese medicinal plant of the family Polygalaceae (Figure 8).

**FIGURE 8:**
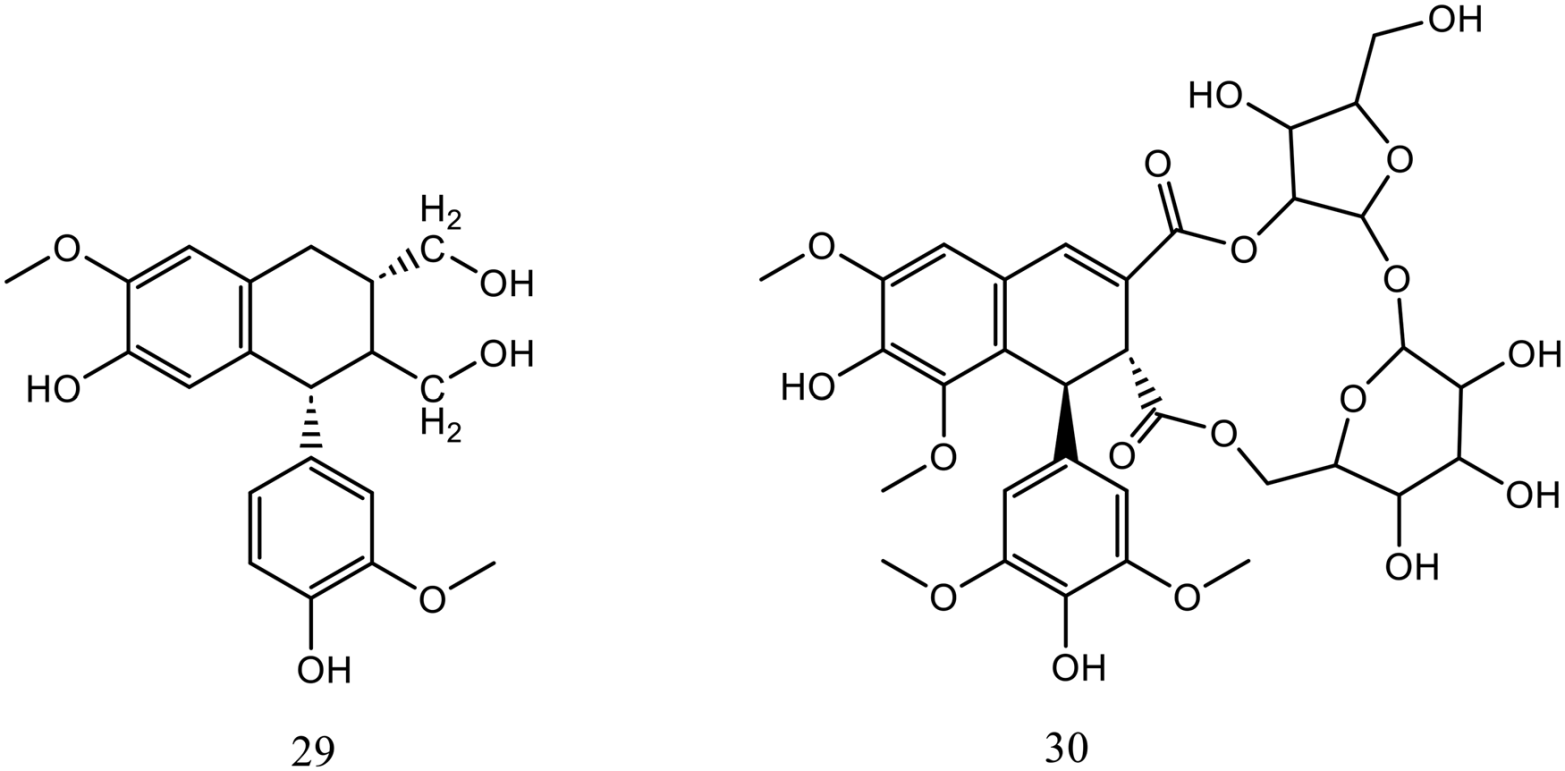
The structures of **29** and **30**.

At present, the research on agricultural and veterinary activities of this subtype lignans is relatively scarce. Existing literature only focuses on their separation, identification, and in vitro antioxidant and anti‐tumor activities in medical models, and there is no systematic bioassay data including in vitro bacteriostasis, insecticidal, herbicide tests, and animal feeding trials.

From the perspective of chemical structure, this kind of lignan has rigid hydrogenated naphthalene ring skeleton and multiple phenolic hydroxyl groups, which can form hydrogen bonds with the target proteins of pathogens and pests, and theoretically have the potential to be developed as green pesticides and veterinary additives. At present, this subtype is a prominent research gap in the field of lignan agricultural application, and large‐scale activity screening needs to be carried out in follow‐up work. Individual lignans such as matairesinol isolated from *Pinus pinea* have weak herbicidal activity against *Portulaca oleracea*, which is the only preliminary agricultural activity record of this subtype [71]. Extracts of fir and spruce knots containing hydroxymatairesinol have inhibitory effects on *Escherichia coli* and wood rot fungi, but only crude extract data without pure compound verification [72].

### 3.5 Mono‐Tetrahydrofuran Lignans

Based on the site of tetrahydrofuran ring cyclization, mono‐tetrahydrofuran lignans can be classified into three structural types—7‐O‐7′, 7‐O‐9′, and 9‐O‐9′—as represented by nectandrin‐B (**31**) from *Myristica fragrans*, as well as olivi‐4′,4′′‐diglucoside (**32**) and olivil (**33**) derived from the bark of *Eucommia ulmoides* Oliv (Figure 9).

**FIGURE 9:**
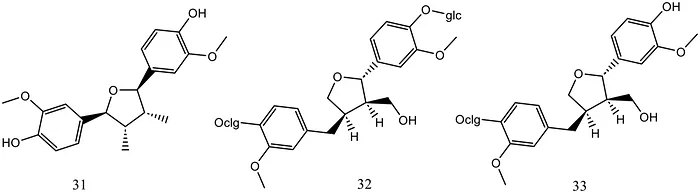
The structures of **31–33**.

Verrucosin (**31**) and nectandrin B (**32**) are representative mono‐tetrahydrofuran lignans, with dual agricultural herbicidal and antifungal activities.

In herbicidal activity, 10 stereoisomers of 3,3′‐dimethoxy‐7,7′‐epoxylignane were fully synthesized in 2014. At 1 × 10^–^^3^ M, (+)‐verrucosin (**34**) almost completely inhibited root growth of *Lolium multiflorum* with inhibition rate close to 100%, superior to diuron; (−)‐verrucosin (**35**) had 94% inhibition rate against *Lactuca sativa* root. Subsequent structural optimization showed that a free phenolic hydroxyl is a necessary group for herbicidal activity, and alkyl substitution will significantly reduce efficacy [73, 74] (Figure 10).

**FIGURE 10:**
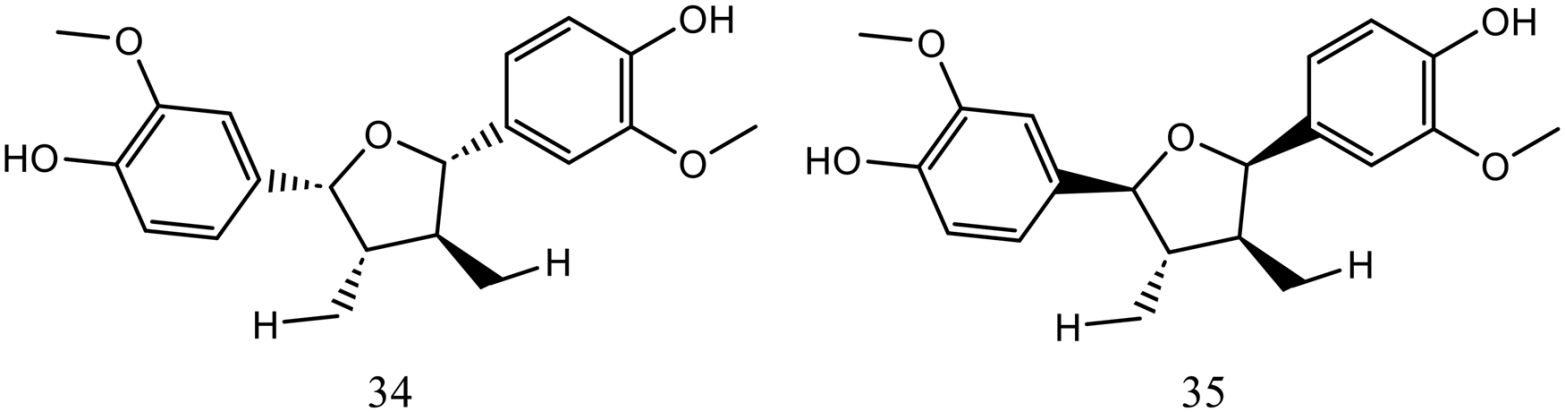
The structures of **34** and **35**.

In the antifungal field, nectandrin B (**31**) separated from *M. fragrans* has broad‐spectrum inhibitory activity against plant pathogenic fungi such as *Magnaporthe grisea* and *Phytophthora infestans*. At 500 mg/L, the field control effect against rice blast and wheat leaf rust reaches 93%, and it also has antibacterial activity against *Burkholderia glumae* [75].

### 3.6 Bis‐Tetrahydrofuran Lignans

Sesamin (**36**) is the core compound of this subtype, mainly applied in veterinary aquaculture and livestock breeding, with partial insecticidal potential against vector mosquitoes.

In laboratory zebrafish models, sesamin (**36**) was reported to alleviate fluoride‐induced thyroid endocrine disruption, reduce embryonic malformation, improve growth performance, and maintain thyroid hormone homeostasis via HPT axis gene regulation [76]. Feeding experiments on *Salmo salar* showed that high‐dose **36** interferes with liver energy metabolism, while appropriate addition can regulate fatty acid composition of muscle tissue [77]. In controlled feeding experiments with weaned piglets, dietary sesamin (**36**) at 0.15% was associated with activation of *Nrf2* and *Wnt/β‐catenin* pathways, improved intestinal barrier function, reduced diarrhea rate and liver fibrosis, and increased the abundance *Lactobacillus* [78]. For *Cyprinus carpio*, **36** has no significant effect on growth performance, and its lipid regulation effect has obvious species specificity [79].

In laboratory larvicidal bioassays, XDA (**38**) from *Z. piperitum* showed LC_50_ values of 0.24–0.27 mg/L against both insecticide‐susceptible and pyrethroid‐resistant *Aedes* strains, and no cross‐resistance was observed under these experimental conditions [80]. In wheat pest resistance research, sesaminol glucoside accumulates in resistant wheat varieties and participates in plant defense response, but its independent insecticidal activity has not been verified [81]. Beyond sesamin, new bis‐tetrahydrofuran (furofuran‐type) lignans continue to be discovered from plant sources; for instance, phillyroside A and phillyroside B, two novel furofuran lignans isolated from the aerial parts of *Forsythia suspensa*, exhibited only weak in vitro free‐radical‐scavenging activity in ABTS assays, suggesting that structural novelty alone does not guarantee bioactivity and that newly isolated bis‐tetrahydrofuran lignans merit systematic agricultural and veterinary screening [82] (Figure 11).

**FIGURE 11:**
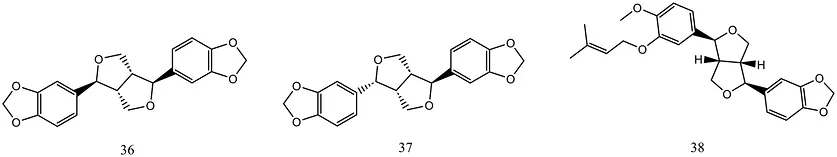
The structures of **36–38**.

In summary, the veterinary applications of sesamin are supported by in vivo animal feeding trials across multiple species (zebrafish, Atlantic salmon, piglets, and common carp), whereas the insecticidal activity of XDA is currently limited to laboratory larval bioassays against mosquito larvae, with no field validation reported to date.

### 3.7 Bicyclooctadiene Lignans

Bicyclooctadiene lignans are a subgroup of classic lignans featuring a unique eight‐membered carbon‐ring fused framework, mostly isolated from *Schisandraceae* medicinal plants. Currently available publications mainly focus on their extraction protocols, in vitro antioxidant assays, and mammalian hepatoprotective pharmacological evaluations. To date, no studies have documented the agricultural bacteriostatic, insecticidal, or herbicidal activities, or the veterinary feeding effects, of bicyclooctadiene lignans. Existing investigations concerning this subtype have been confined to its isolation from *Schisandra* plants and to in vitro pharmacological tests of antioxidant activity and hepatoprotection. Given the multiple phenolic hydroxyl groups in its structure, this subtype is hypothesized to hold potential for agricultural freshness preservation and veterinary antioxidant applications; nevertheless, no experimental data are currently available to support such applications, leaving a significant gap for subsequent research.

For this structural class, no in vitro or in vivo agro‐veterinary bioactivity data have been reported in the peer‐reviewed literature. The present review includes this subtype solely to document its current research gap; therefore, no experimental validation level is assigned to any compound within this category.

## 4 Neolignans

The following subsections summarize the reported agro‐veterinary activities of neolignans. Notably, the validation levels vary widely across the three structural subtypes: benzofuran and biphenyl neolignans have been tested from in vitro to in vivo (pot/animal) levels, whereas benzodioxane neolignans lack any agro‐veterinary data. Neolignans are phenylpropane dimers connected by non‐β′─β bonds, divided into benzofuran, benzodioxane, and biphenyl three subtypes. At present, benzofuran and biphenyl neolignans have abundant agricultural and veterinary research data, while benzodioxane neolignans lack relevant activity reports.

### 4.1 Benzofuran Neolignans

Magnolol (**39**), piperenone (**40**), and licarin A (**41**) are typical benzofuran neolignans, with agricultural antifungal, insecticidal, and veterinary antibacterial synergistic effects.

Agricultural antifungal activity: **39**, separated from *Caryodaphnopsis baviensis*, has an IC_50_ of 4.5 µg/mL against *Alternaria porri*, with broad‐spectrum inhibitory activity against *Alternaria brassicicola*, *M. oryzae*, and other pathogenic fungi. At 500 µg/mL, the potted control effect against onion purple spot disease exceeds 80%, and the mechanism is to inhibit the mitochondrial respiratory chain complex to block pathogen energy metabolism [83].

In laboratory bioassays, piperenone (**42**) from *Piper kadsura* caused 100% lethality against *A. albopictus* larvae at 0.01 mg/mL. A Pickering emulsion formulation improved its larvicidal activity and showed low toxicity to non‐target organisms (bees and medaka) in laboratory assessments [84].

Veterinary antibacterial synergism: **41**, pibeneolignan I (**43**) and other compounds isolated from *Piper betle* can significantly reduce the MIC of norfloxacin against norfloxacin‐resistant *S. aureus* by inhibiting the NorA efflux pump, with FICI ≤0.5 showing obvious synergistic antibacterial effect, and no cytotoxicity at effective concentration, which can be developed as a veterinary antibiotic synergist [85] (Figure 12).

**FIGURE 12:**
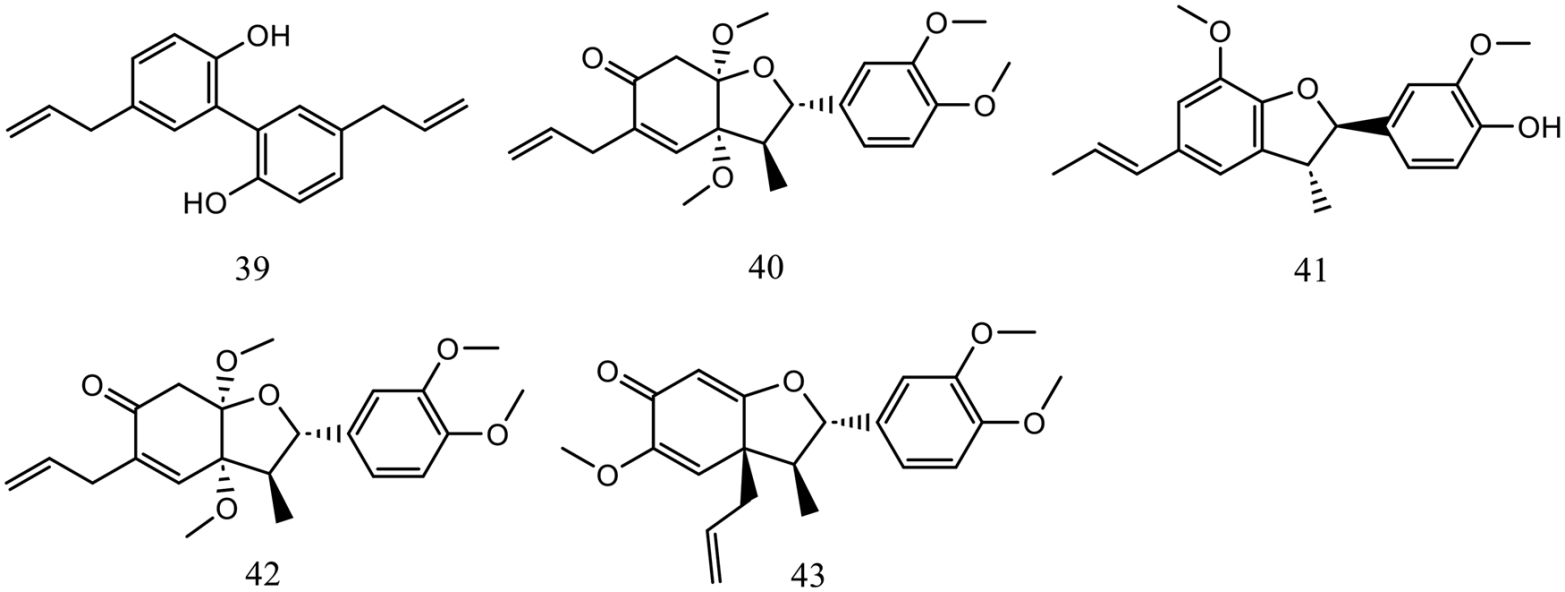
The structures of **39–43**.

Among benzofuran neolignans, magnolol has progressed from in vitro antifungal screening to in vivo pot trials (onion purple blotch, >80% control efficacy). Piperenone's larvicidal activity remains at the laboratory in vivo level, while licarin A's synergistic antibacterial effects are confined to in vitro checkerboard assays. No field trial data are available for this subtype.

### 4.2 Benzodioxane Neolignans

In all collected literature, there are no experimental reports on agricultural antifungal, insecticidal, herbicidal activity and veterinary feeding, anti‐parasitic effects of benzodioxane neolignans. The existing research only covers their separation, identification, and in vitro anti‐inflammatory activity in human cell models. Compared with benzofuran neolignans with a similar skeleton, the benzodioxane heterocycle has stronger lipophilicity, which is theoretically easier to penetrate the cell membrane of pathogens and insect epidermis, but there is no biological activity data to verify this inference at present, which is a research blank that needs to be filled.

### 4.3 Biphenyl Neolignans

Magnolol (**44**) and honokiol (**45**) from *Magnolia officinalis* are the most studied biphenyl neolignans, covering agricultural fungus, pest control, and multi‐scenario veterinary applications such as poultry and aquatic animals.

Agricultural antifungal field: **44** has strong inhibitory activity against *Fusarium oxysporum* f. sp. cubense causing banana fusarium wilt and *Trichoderma hamatum* causing sweet potato rot. It destroys the pathogen cell membrane, interferes with energy metabolism, and has no phytotoxicity to crops at effective concentration; its brominated derivative has an EC_50_ as low as 2.86 µg/mL against *Botrytis cinerea* [86, 87, 88].

Agricultural insecticidal and acaricidal field: **44** and **45** are reversible mixed inhibitors of insect AChE, with LC_50_ of 0.137 Mm against *Nilaparvata lugens*, superior to chlorpyrifos; azo‐modified derivatives have corrected mortality over 63% against *M. separata* larvae [89, 90]. A magnolol derivative showed anti‐*Ichthyophthirius* activity in aquarium‐scale laboratory trials, with low embryotoxicity to zebrafish in laboratory assays [91].

Veterinary application field: Adding **44** to laying hen feed can alleviate fatty liver hemorrhagic syndrome, reduce serum lipid level and liver lipid deposition by regulating PPAR pathway; it can improve egg quality and intestinal tight junction protein expression in late laying hens [92, 93]. For broilers infected with *Salmonella pullorum*, **44** supplementation improves intestinal villus morphology, increases the abundance of beneficial *Lactobacillus*, and reduces inflammatory factor expression [94]. Furthermore, a meta‐analysis integrating 18 studies reported that dietary microalgae supplementation did not significantly affect the production performance (body weight gain, daily gain, feed intake, and feed conversion ratio) or the blood hematological and serum metabolite parameters of broilers, whereas it significantly increased spleen weight (*p*<0.001) and elevated the levels of omega‐3 fatty acids (C22:5n‐3, C22:6n‐3, and total omega‐3) while decreasing certain omega‐6 fatty acids and the omega‐6/omega‐3 ratio in breast meat; accordingly, microalgae supplementation can effectively reduce the n‐6/n‐3 fatty acid ratio of broiler meat, which is advantageous for the health of broilers and consumers [95]. The biphenyl neolignan skeleton likewise continues to furnish structurally novel derivatives, as exemplified by gymnothebutyllignans A and B, two new biphenyl butyl neolignans characterized from the endemic Chinese medicinal plant *Gymnotheca involucrata* (Saururaceae); no agricultural or veterinary bioactivity has been reported for these compounds to date, representing a further screening opportunity within the biphenyl subtype [96] (Figure 13).

**FIGURE 13:**
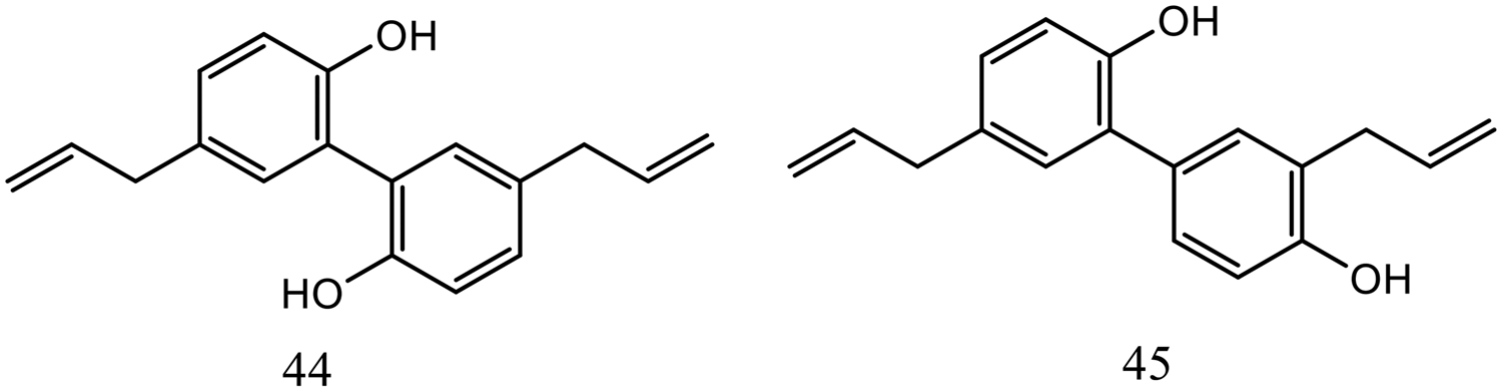
The structures of **44** and **45**.

For biphenyl neolignans (magnolol and honokiol), the available evidence spans in vitro antifungal assays, laboratory in vivo insecticidal bioassays, and in vivo animal feeding trials in poultry. However, no greenhouse pot trials or open‐field efficacy studies have been reported for these compounds. The anti‐Ichthyophthirius activity of magnolol derivatives was evaluated in in vitro parasite assays coupled with in vivo zebrafish embryo toxicity observations, which do not constitute field aquaculture validation.

## 5 Oligolignans

Oligolignans refer to polymers formed by three or more phenylpropane units. The collected literature only involves the separation of trimeric lignans from Picea and Abies wood knots, and only in vitro DPPH antioxidant test data are available. There is no systematic research on agricultural bacteriostasis, insecticidal, or herbicidal activity and livestock/aquatic animal feeding experiments.

The existing data show that oligolignans have stronger free radical scavenging ability than dimeric lignans, but their high molecular weight leads to poor solubility and difficult plant extraction. Multiple hydrogen‐bonding sites brought by polymerization make them theoretically have multi‐target synergistic agricultural and veterinary activity. At present, there is a lack of pure compound activity evaluation, in vivo pot and field trials, as well as long‐term animal feeding safety verification, which is one of the main weak links in lignan agricultural research.

## 6 Complex Lignans

Among complex lignans, only flavonolignans (silymarin) have reported bioactivity data relevant to veterinary applications. Coumarin‐lignan and terpenoid‐lignan hybrids have no agro‐veterinary activity records and are thus not assigned validation levels.

Complex lignans are hybrid compounds formed by a lignan skeleton combined with flavonoids, coumarins, and other secondary metabolites. Flavolignans represented by silymarin (**46**) have relatively sufficient agricultural and veterinary research data, while coumarin‐lignan and terpenoid‐lignan hybrid derivatives have no relevant activity records in the collected literature (Figure 14).

**FIGURE 14:**
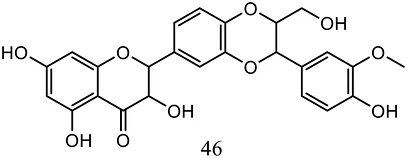
The structures of **46**.

### 6.1 Flavolignans (Silymarin)

In 2024, Khorsand et al. [97] demonstrated that **46**, a flavonolignan from *Silybum marianum*, serves as an effective cryoprotective antioxidant in stallion semen preservation.

Arabian stallion semen samples were frozen in extenders supplemented with 0–100 µg/mL **46** and assessed post‐thaw for motility, mitochondrial membrane potential (MMP), plasma membrane integrity (PMI), lipid peroxidation (LPO), DNA fragmentation (DFI), and apoptosis. **46** at 25–100 µg/mL significantly improved viability, motility, and MMP while reducing LPO, DFI, and apoptosis (pANOVA, < 0.05). The 100 µg/mL treatment yielded the optimal effects: total motility increased from 35.8% to 68.5%, progressive motility from 13.4% to 28.6%, and DFI decreased from 25.8% to 10.6%. PMI was significantly enhanced at 75–100 µg/mL. Mechanistically, **46** scavenges reactive oxygen species, protects membrane lipids from peroxidation, maintains mitochondrial function, and inhibits apoptosis. This ex‐vivo study highlights silymarin's potential as a natural antioxidant additive in laboratory‐scale semen preservation models for stallions, improving post‐thaw sperm quality under the experimental conditions tested. In Vivo feeding trials or field artificial insemination studies are required to confirm its practical veterinary utility.6.2 Coumarin‐lignan & Terpenoid‐lignans.

It should be emphasized that the silymarin data presented here are derived from an ex vivo semen preservation model (stallion spermatozoa processed in vitro after collection), which is classified as an in vitro assay for the purpose of experimental validation. These findings demonstrate proof‐of‐concept for reproductive biotechnology but have not been validated through in vivo breeding trials or field artificial insemination studies.

### 6.2 Coumarin‐Lignan & Terpenoid‐Lignans

For coumarin‐lignan and terpenoid‐lignan hybrids, no literature reporting agricultural or veterinary bioactivity was identified in our search. These hybrid compounds have only been characterized chemically or evaluated in human‐cell‐based pharmacological assays. Therefore, no experimental validation level is assigned, and these structural classes represent a complete research void for agro‐veterinary applications.

## 7 Cross‐Skeleton Comparative Analysis

To provide a visual overview of how far each lignan subtype has progressed along the experimental validation pipeline, we present the following summary table (Table 1). The validation levels are defined as: in vitro (cell‐free or isolated pathogen/parasite assays), in vivo–laboratory (whole‐organism tests under controlled lab conditions), in vivo–pot (greenhouse/potted plant trials), in vivo–animal (live animal feeding or infection models), and field trial (open‐field or commercial‐scale validation).

**TABLE 1:** Experimental validation levels of reported agro‐veterinary activities for lignan subtypes.

Lignan subtype	Representative compound	In vitro	In vivo (laboratory)	In vivo (pot/greenhouse)	In vivo (animal feeding)	Field trial
Dibenzylbutane	NDGA	✓ (LOX, antimicrobial)	✓	✓	—	✓ (Fruit)
Dibenzylbutane	SDG	✓ (Antioxidant)	—	—	✓ (Dogs, rats, hens, goats)	—
Dibenzylbutyrolactone	Arctigenin	✓ (Cell, parasite)	✓ (Larvae)	—	—	—
Arylnaphthalene lactone	Podophyllotoxin	✓	✓ (Larvae)	—	—	✓ (Rice blast, 1 study)
Mono‐tetrahydrofuran	Nectandrin B	✓	—	—	—	✓ (Fungal)
Bis‐tetrahydrofuran	Sesamin	✓	✓ (Larvae)	—	✓ (Fish, pigs)	—
Benzofuran neolignan	Magnolol	✓ (Antifungal)	✓ (Larvae)	✓ (Pot)	—	—
Biphenyl neolignan	Magnolol/honokiol	✓ (Antifungal)	✓ (Insect)	—	✓ (Poultry)	—
Flavolignan	Silymarin	✓ (Semen)	—	—	—	—

*Note*: Checkmark (✓) denotes confirmed presence; em dash (—) denotes not detected (blank).

Significant uneven distribution of research efforts exists across lignan sub‐classes. Classic lignans (dibenzylbutane, dibenzylbutyrolactone, arylnaphthalene‐lactone) possess abundant in vitro bioassay datasets covering insecticidal, antifungal, fruit‐ripening‐regulation, and veterinary feed‐additive activities. Their major bottlenecks are low natural abundance, complex total‐synthesis routes, and unsatisfactory water solubility. Among them, arylnaphthalene‐lactone (podophyllotoxin‐series) shows outstanding insecticidal/acaricidal potency after semi‐synthetic halogen or heterocyclic modification, which ranks high as pesticide lead candidates.

Neolignans: Biphenyl‐subtypes (magnolol/honokiol) own complete datasets including in vitro, pot test, and animal feeding results; benzofuran neolignans show promising fungicidal and synergistic‐antibiotic effects; benzodioxane neolignans remain almost blank without agro‐veterinary phenotype data.

Oligolignans and most complex‐lignan hybrids (apart from flavolignan silymarin) are poorly explored. Higher‐molecular‐weight oligolignans have strong antioxidant capacity, yet suffer from poor water solubility and difficulties in natural isolation, hindering further bio‐evaluation.

From activity spectrum perspective: Insecticidal/acaricidal: arylnaphthalene lactone > dibenzylbutyrolactone > biphenyl neolignans; antifungal: Dibenzylbutane derivatives, biphenyl neolignans; veterinary antioxidant/intestinal‐regulation: Dibenzylbutane SDG, flavolignan silymarin; herbicidal activity: Only limited data from mono‐tetrahydrofuran lignans.

This cross‐skeleton comparison highlights that most subtypes still lack systematic multi‐parameter screening, and structure–activity rules cannot be generalized across different carbon frameworks.

Based on the combined evaluation of potency, available mechanism data, and in vivo validation, several lignans emerge as the most promising leads for agro‐veterinary development: Podophyllotoxin and its semi‐synthetic derivatives rank highest for insecticidal/acaricidal development due to their potent EC_50_/LC_50_ values and multiple modification sites; NDGA is the most validated compound for pre‐/post‐harvest fruit preservation via NCED inhibition; MAGNOLOL and honokiol exhibit the most comprehensive veterinary application portfolio, covering poultry gut health and aquaculture parasite control; Arctigenin shows the greatest specificity and therapeutic index for aquatic antiparasitic applications.

The molecular targets and pathways underlying the agro‐veterinary activities of representative lignans are summarized schematically in Figure 15.

**FIGURE 15:**
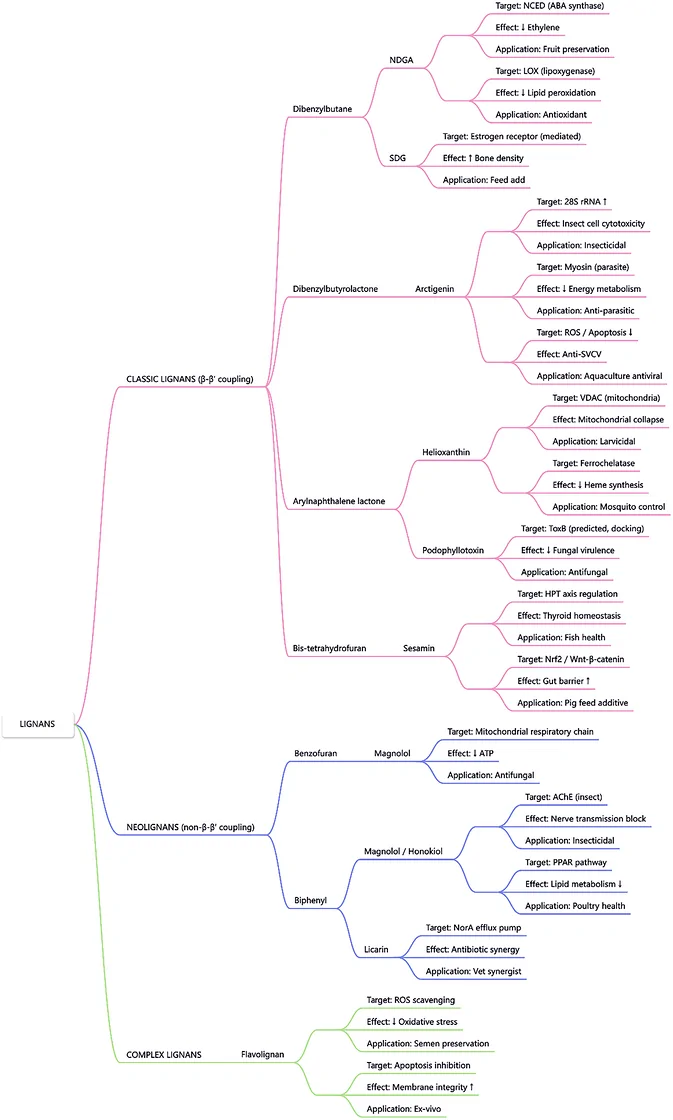
Schematic diagram linking lignan subclasses with their reported molecular targets, affected pathways, and corresponding biological effects in agricultural and veterinary contexts.

## 8 Toxicological and Environmental Considerations

Beyond their reported bioactivity, the practical deployment of lignans as agrochemicals and feed additives is critically governed by their toxicological profiles and environmental fate. However, systematic data on phytotoxicity, mammalian toxicity, effects on non‐target organisms, and environmental persistence remain scarce for most lignan scaffolds, and these knowledge gaps constitute a major bottleneck for industrial translation.

### 8.1 Phytotoxicity

The dual role of lignans as both bioactive leads and potential phytotoxicants deserves particular attention. The herbicidal activity of verrucosin enantiomers, which nearly completely inhibited root growth of *Lolium multiflorum* and *Lactuca sativa* at 1 × 10^–^^3^ M, directly reflects phytotoxic effects that are desirable for weed suppression but may also raise concerns for non‐target vegetation [73, 74]. Conversely, magnolol displayed no phytotoxicity to crops at effective antifungal concentrations [86, 87, 88], indicating that phytotoxicity is scaffold‐ and dose‐dependent. Systematic evaluation of crop selectivity, including root and shoot growth‐inhibition bioassays against representative crop and weed species, is therefore required before herbicidal application.

### 8.2 Mammalian Toxicity

Although lignans are generally regarded as low‐toxicity natural products, several representative compounds exhibit pronounced mammalian toxicity. NDGA, extensively studied for fruit preservation, was removed from the U.S. FDA Generally Recognized as Safe (GRAS) list in 1968 on the basis of evidence of hepatotoxicity and nephrotoxicity; oxidation of its catechol rings to reactive ortho‐quinones, which form protein and DNA adducts and deplete glutathione, is regarded as the underlying mechanism [98]. Podophyllotoxin is highly toxic to mammals and unsuitable for oral use; it induces hepatic and intestinal toxicity in mice, and accidental ingestion has been associated with severe neurological, hematological, and gastrointestinal disturbances [99, 100]. By contrast, magnolol and honokiol exhibit comparatively low acute oral toxicity in rodents and are generally well tolerated at moderate doses, although their long‐term safety margins remain incompletely defined [101]. These contrasting profiles underscore that ‘natural origin’ does not guarantee safety, and compound‐specific toxicological evaluation is indispensable.

### 8.3 Non‐Target Organisms

The available evidence on non‐target effects is fragmentary but informative. Several lignan derivatives displayed encouraging selectivity, including sulfonylated helioxanthin derivatives with reduced toxicity to bees and non‐target plants, a pickering emulsion of piperenone with low toxicity to bees and medaka, and magnolol derivatives with low embryotoxicity to zebrafish [66, 69, 84, 91]. However, adverse aquatic effects have also been documented: honokiol caused liver hemorrhage and gill tissue swelling and necrosis in zebrafish at 0.6 mg/L [102], and a crude water extract of *Schisandra chinensis* fruits, whose dominant active principles are lignans (schisandrin), exhibited high acute toxicity to *Daphnia magna* (EC_50_ = 0.0448 mg/L after 24 h) [103]. These findings caution against a blanket assumption of environmental safety and highlight the need for systematic testing against bees, aquatic invertebrates, fish, and soil organisms.

### 8.4 Environmental Persistence and Biodegradation

For botanical pesticides, microbial‐mediated degradation is generally considered the predominant dissipation pathway in soil, with accelerated degradation in non‐sterilized soils, complemented by abiotic processes such as photolysis and hydrolysis [104]. Degradation rates are strongly influenced by soil physicochemical properties, including clay and organic‐matter content, pH, moisture, and temperature, and some degradation metabolites may be more persistent or more toxic than the parent compounds [104]. To date, systematic studies on the soil half‐life, transformation pathways, and metabolic fate of lignan‐type agrochemicals are almost entirely lacking, representing a critical gap for environmental risk assessment.

Collectively, the toxicological and environmental dimensions of lignans remain the least understood aspects of their agro‐veterinary development. Future work should prioritize standardized phytotoxicity, mammalian and non‐target toxicity testing, as well as degradation kinetics and environmental fate studies, so that promising lignan leads can be advanced with a complete safety and ecological profile.

## 9 Formulation and Delivery Strategies

A fundamental obstacle to the practical use of lignans in agriculture and animal husbandry is their poor aqueous solubility and limited stability, which constrain field efficacy and oral bioavailability. Recent formulation research has addressed this bottleneck through cyclodextrin complexation, nano‐based carrier systems, prodrug design, and stimulus‐responsive delivery platforms, offering promising routes for translating promising lignan leads into commercially viable products.

### 9.1 Cyclodextrin Complexation

Cyclodextrin (CD) inclusion is one of the most straightforward approaches to solubilize hydrophobic lignans without covalent modification. For podophyllotoxin, complexation with a biotin‐conjugated β‐cyclodextrin (mono‐6‐biotin‐amino‐6‐deoxy‐β‐cyclodextrin) prepared by freeze‐drying increased the aqueous solubility by approximately ninefold relative to the free drug and afforded a stability constant (*K*_s_ = 415.29 M^–^^1^) 3.2 times that of the parent β‐CD complex, while also improving cellular uptake and antitumor potency [105]. CD‐based inclusion thus represents a facile and scalable strategy to improve both solubility and stability of lignan agrochemicals.

### 9.2 Nano‐Based Carriers for Solubility and Bioavailability

Nanosuspensions, polymeric micelles, and solid nanoparticles have been developed to enhance lignan dissolution and absorption. Magnolol‐loaded mixed micelles and nanosuspensions prepared with Soluplus and Poloxamer 188 increased gastrointestinal absorption by 2.85‐ and 2.27‐fold, respectively, relative to the free drug [106]. Self‐assembling lecithin‐based mixed polymeric micelles (lbMPMs) stabilized honokiol and magnolol in biological media and significantly improved their oral bioavailability (absolute bioavailability of 4.8% for honokiol and 20.1% for magnolol with the optimized NaDOC formulation) [107]. In parallel, honokiol nanoparticles fabricated by liquid antisolvent precipitation converted the crystalline drug into an amorphous state, raising its dissolution rate by approximately 20.4‐ and 26.2‐fold in artificial gastric and intestinal fluids, respectively, with a 6.52‐fold greater AUC in vivo [108]. For sesamin, a self‐nanoemulsifying drug delivery system (SNEDDS) increased intestinal permeability by more than 3‐fold and raised the absolute bioavailability from 0.3% to 4.4% (approximately 12.9‐fold relative bioavailability) [109]. These nano‐platforms collectively demonstrate that dissolution‐limited absorption of hydrophobic lignans can be substantially overcome by well‐designed carrier systems.

### 9.3 Prodrug and Bioconjugation Strategies

Covalent modification offers an additional route to improve aqueous solubility and controlled activation. A recent review systematically summarized bioconjugation and nanosystem strategies for podophyllotoxin, including prodrug construction that maintains the pharmacophore while enhancing solubility, stability, and target selectivity [110]. Prodrug and conjugation approaches are particularly attractive for lignans bearing phenolic hydroxyls, which serve as versatile handles for reversible derivatization and enable triggered release at target sites.

### 9.4 Field Delivery: Nanocarriers for Sustained and Stimulus‐Responsive Release

Beyond solubilization, advanced nanocarriers are being engineered for field‐relevant delivery of botanical pesticides. Bio‐based lignin nanocarriers loaded with fungicides have been shown to protect active ingredients, provide sustained release, and degrade upon pathogen‐secreted ligninolytic enzymes, enabling single trunk‐injection treatment against wood diseases such as Esca in grapevine [111]. Enzyme‐responsive lignin nanoparticles loaded with pyraclostrobin achieved a 2.92‐fold improvement in photostability relative to the commercial suspension concentrate, with enzyme‐triggered release and excellent control efficacy against Botrytis cinerea on strawberry [15]. Lignin‐based carriers are especially attractive for lignan‐type agrochemicals because their polyphenolic structure is compatible with hydrophobic payloads and their biodegradability aligns with the green‐agriculture agenda.

Collectively, the emerging formulation toolbox—spanning cyclodextrin inclusion, nanosuspensions, mixed micelles, SNEDDS, prodrug design, and lignin‐based nanocarriers—provides complementary solutions to the solubility, stability, bioavailability, and field‐delivery limitations of lignans. Integrating these approaches with the structure–activity optimization and safety assessment discussed above will be essential to advance lignan‐derived agrochemicals and feed additives from laboratory proof‐of‐concept toward practical applications.

## 10 Retrospective and Outlook

From the perspective of chemical skeletons, this systematic review of lignan research over the 2001–2026 period reveals a marked imbalance in research progress between agricultural and veterinary applications.

Among classic lignans, the dibenzylbutane (e.g., NDGA, SDG), dibenzylbutyrolactone (e.g., arctigenin), and arylnaphthalene lactone (e.g., podophyllotoxin) subtypes have been extensively investigated, with reported applications spanning fruit preservation, antifungal and pest control, aquatic antiparasitic treatments, and poultry feed additives. In contrast, mono‐ and bis‐tetrahydrofuran lignans possess only limited data on agricultural insecticidal and veterinary antioxidant activities, whereas the aryl tetrahydronaphthalene and bicyclooctadiene subtypes remain virtually unexplored. For neolignans, the biphenyl (e.g., magnolol, honokiol) and benzofuran subtypes are well characterized, with complete datasets from in vitro, pot, and animal feeding trials, yet no agricultural or veterinary activity has been reported for benzodioxane neolignans. Oligolignans and other complex lignans (excluding flavonolignans) have received very little attention.

From an application‐oriented perspective, research in the agricultural domain has predominantly focused on antifungal, insecticidal, and fruit‐ripening regulation activities, while herbicidal data are comparatively scarce, with only a small number of tetrahydrofuran lignans having been evaluated for growth inhibition. In the veterinary domain, current efforts center on antioxidant properties, intestinal microecological modulation, anti‐inflammatory effects, and mycotoxin detoxification as feed additives. Notably, aquatic antiparasitic and antiviral lignan derivatives have emerged in recent years as a promising and rapidly growing research line.

Regarding structural optimization strategies, the mainstream modification approaches for active lignans include halogenation, esterification, etherification, heterocyclic fusion, and stereoisomer transformation. The introduction of halogen atoms, imidazole, or isoxazoline moieties can significantly improve EC_50_/LC_50_ values, while stereoconfiguration plays a critical role in determining the selectivity of lignans toward insect versus mammalian cells.

### 10.1 Critical Research Gaps

Despite the progress summarized above, several fundamental deficiencies persist. First, the distribution of research efforts across different chemical skeletons remains highly uneven, with the vast majority of studies concentrated on a limited number of compounds—magnolol, podophyllotoxin, NDGA, sesamin, and a few other frequently investigated molecules—whereas most minor subtypes have not even undergone basic activity screening. Moreover, natural product mining has been largely confined to a narrow range of plant families, leaving considerable chemodiversity untapped. Second, mechanistic investigations remain superficial for all but a handful of representative lignans. While molecular targets such as NCED, VDAC, ferrochelatase, ACh, and the NorA efflux pump have been identified in isolated cases, the vast majority of lignans have only been evaluated for phenotypic inhibition rates, with no accompanying analysis of intracellular metabolic pathways or protein‐binding mechanisms. This lack of mechanistic clarity severely hampers targeted molecular optimization. Third, the current testing systems are notably incomplete. Most bioactivity evaluations are confined to in vitro assays using cultured cells or isolated pathogens and insects; data from pot experiments, field efficacy trials, long‐term livestock feeding safety studies, environmental degradation profiles, and non‐target organism toxicity assessments remain scarce, rendering current findings insufficient to support industrial development of lignan‐based pesticides and feed additives. Fourth, formulation research has been conspicuously neglected, with few studies addressing the poor water solubility of lignans—a critical bottleneck for field application—and minimal progress in developing sustained‐release systems, microemulsions, or other green formulations.

### 10.2 Future Directions

To address the aforementioned limitations, several priority areas merit focused attention. (1) Comprehensive resource screening. Systematic agricultural and veterinary activity evaluations should be extended to currently unexplored subtypes, including aryl tetrahydronaphthalene lignans and benzodioxane neolignans, while broader screening of endemic native plants may uncover novel active components. (2) Target‐oriented structural modification. Future derivatization should be guided by mechanism‐based design, integrating molecular docking and multi‐omics approaches to elucidate the precise targets of highly active lignans, thereby enabling the rational design of high‐efficacy, low‐toxicity derivatives. Particular emphasis should be placed on optimizing herbicidal lead compounds, which remain critically underrepresented. (3) Comprehensive in vivo and environmental evaluation. The research pipeline must be extended to include field pesticide efficacy trials, long‐term livestock feeding studies, and full ecological safety assessments, with systematic evaluation of residue profiles, degradation kinetics, and toxicity to non‐target organisms such as bees and fish. (4) Formulation development. Investment in green formulation technologies—including water‐soluble microcapsules and Pickering emulsions—is urgently needed to overcome the poor solubility characteristic of polyphenolic lignans, facilitating their transition from laboratory compounds to commercially viable agrochemicals and feed additives. (5) Expansion of application scenarios. Future research should strengthen investigations into lignan‐based herbicides and multifunctional veterinary additives that combine antibacterial and anti‐mycotoxin properties, thereby aligning with the growing demand for green agriculture and antibiotic‐free animal husbandry.

## 11 Conclusion

In this comprehensive review, we categorized lignans and neolignans by carbon‐skeleton origin and systematically summarized their agricultural plant‐protection and veterinary‐additive bioactivities. Dibenzylbutane, dibenzylbutyrolactone, arylnaphthalene‐lactone classic lignans, and biphenyl‐type neolignans constitute the most intensively investigated subclasses, showing promising potential as botanical fungicides, insecticides, fruit‐ripening regulators, and natural livestock‐feed supplements. Nevertheless, large‐scale knowledge gaps persist for multiple structural groups: bicyclooctadiene lignans, benzodioxane‐neolignans, oligolignans, and most hybrid complex lignans still lack systematic phenotypic bio‐evaluation. Most reported lignan bio‐data are limited to in vitro screening; pot tests, field efficacy, non‐target‐organism toxicity, and long‐term animal‐safety datasets remain scarce. The accumulated structure‐activity evidence indicates that halogen substitution, suitable heterocyclic‐fragment introduction, and stereochemical configuration are decisive factors for improving the pesticidal potency of lignan leads. Phenolic‐hydroxyl groups are essential for antioxidant and partial antimicrobial performance. Further research should prioritize filling the screening gaps of under‐explored skeletons, mechanism‐oriented structural modification, complete in vivo environmental safety assessment, and green‐formulation development, to promote lignan natural products translating from laboratory‐scale bio‐assays toward practical agro‐veterinary utilization.

## Author Contributions

The corresponding authors confirm that all the authors have made substantial contributions to this manuscript. **Kaida Li**: investigation, formal analysis, writing – original draft. **Huawen Wang**: conceptualization, data curation, writing – review & editing. **Xiangjie Luo**: resources, software. All authors have read and agreed to the published version of the manuscript.

## Funding

This work was supported by a grant from Yunnan University Graduate Students Research and Innovation Project (ZC‐252513371).

## Conflicts of Interest

The authors declare no conflicts of interest.

## Data Availability

The data that support the findings of this study are available from the corresponding author upon reasonable request.
